# Changes in a Digital Type 2 Diabetes Self-management Intervention During National Rollout: Mixed Methods Study of Fidelity

**DOI:** 10.2196/39483

**Published:** 2022-12-07

**Authors:** Jack S Benton, Sarah Cotterill, Rhiannon E Hawkes, Lisa M Miles, David P French

**Affiliations:** 1 Manchester Centre for Health Psychology Division of Psychology and Mental Health University of Manchester Manchester United Kingdom; 2 Centre for Biostatistics Division of Population Health, Health Services Research & Primary Care University of Manchester Manchester United Kingdom

**Keywords:** type 2 diabetes, Healthy Living, digital interventions, behavior change, self-management, fidelity, implementation, mixed methods, mobile phone

## Abstract

**Background:**

“Healthy Living for People with type 2 Diabetes (HeLP-Diabetes)” was a theory-based digital self-management intervention for people with type 2 diabetes mellitus that encouraged behavior change using behavior change techniques (BCTs) and promoted self-management. HeLP-Diabetes was effective in reducing HbA1c levels in a randomized controlled trial (RCT). National Health Service (NHS) England commissioned a national rollout of HeLP-Diabetes in routine care (now called “Healthy Living”). Healthy Living presents a unique opportunity to examine the fidelity of the national rollout of an intervention originally tested in an RCT.

**Objective:**

This research aimed to describe the Healthy Living BCT and self-management content and features of intervention delivery, compare the fidelity of Healthy Living with the original HeLP-Diabetes intervention, and explain the reasons for any fidelity drift during national rollout through qualitative interviews.

**Methods:**

Content analysis of Healthy Living was conducted using 3 coding frameworks (objective 1): the BCT Taxonomy v1, a new coding framework for assessing self-management tasks, and the Template for Intervention Description and Replication. The extent to which BCTs and self-management tasks were included in Healthy Living was compared with published descriptions of HeLP-Diabetes (objective 2). Semistructured interviews were conducted with 9 stakeholders involved in the development of HeLP-Diabetes or Healthy Living to understand the reasons for any changes during national rollout (objective 3). Qualitative data were thematically analyzed using a modified framework approach.

**Results:**

The content analysis identified 43 BCTs in Healthy Living. Healthy Living included all but one of the self-regulatory BCTs (“commitment”) in the original HeLP-Diabetes intervention. Healthy Living was found to address all areas of self-management (medical, emotional, and role) in line with the original HeLP-Diabetes intervention. However, 2 important changes were identified. First, facilitated access by a health care professional was not implemented; interviews revealed this was because general practices had fewer resources in comparison with the RCT. Second, Healthy Living included an additional structured web-based learning curriculum that was developed by the HeLP-Diabetes team but was not included in the original RCT; interviews revealed that this was because of changes in NHS policy that encouraged referral to structured education. Interviewees described how the service provider had to reformat the content of the original HeLP-Diabetes website to make it more usable and accessible to meet the multiple digital standards required for implementation in the NHS.

**Conclusions:**

The national rollout of Healthy Living had good fidelity to the BCT and self-management content of HeLP-Diabetes. Important changes were attributable to the challenges of scaling up a digital intervention from an RCT to a nationally implemented intervention, mainly because of fewer resources available in practice and the length of time since the RCT. This study highlights the importance of considering implementation throughout all phases of intervention development.

## Introduction

### Background

Type 2 diabetes mellitus (T2DM) is one of the most common long-term conditions worldwide [1]. T2DM can lead to a range of health complications, but many of these complications can be prevented if individuals effectively self-manage their condition through healthy eating, physical activity, blood glucose monitoring, medication adherence, problem-solving skills, coping skills, and risk-reduction behaviors [2]. However, performing effective self-management is demanding and influenced by many contextual factors (eg, family, financial status, and community environment) [3], which means it can be difficult to meet the challenges of self-management without support. Self-management interventions can give people the knowledge, skills, and confidence to improve self-management through education, training, and support. Self-management interventions for people with T2DM are recommended by the UK National Institute for Health and Care Excellence for all people diagnosed with T2DM [4].

Self-management interventions for people with T2DM are typically delivered through face-to-face or group-based courses [5-7]. Although these interventions can improve clinical and psychosocial outcomes in people with T2DM [8] and are cost-effective [9], attendance can be extremely low. For example, data from the UK National Diabetes Audit suggest that just 7% of people newly diagnosed with T2DM who were offered structured education were recorded as attending within 1 year of diagnosis [10]. As an alternative, digital interventions (via digital technologies such as websites or smartphones) have the potential to be more convenient for patients as they can be delivered at scale in multiple locations, which also consumes fewer primary care resources. Mounting evidence suggests that digital self-management interventions can improve glycemic control (HbA1c) in people with T2DM [11-13].

A digital self-management intervention that has demonstrated effectiveness is Healthy Living for People With Type 2 Diabetes (HeLP-Diabetes), mainly consisting of a theory- and evidence-based website. In a randomized controlled trial (RCT) in 21 primary care practices in England, HeLP-Diabetes led to a significant, albeit modest, reduction in HbA1c levels of 0.24% (95% CI −0.44% to –0.049%; *P*=.01) at 12 months and was found to be cost-effective [14]. The HeLP-Diabetes website contained information about understanding and treating T2DM, behavior change modules, self-help tools, self-assessment quizzes, videos from people with T2DM, a moderated web-based forum, and an electronic health record. Facilitated access with a practice nurse was provided as part of the HeLP-Diabetes intervention, which consisted of an introductory training session with the practice nurse. Follow-up telephone calls were offered to support patients with using the website.

HeLP-Diabetes was originally designed as an unstructured digital intervention that patients could access without following a linear pathway, and it was this intervention that was tested in the RCT; this study focuses on this intervention. However, in 2013, general practitioners in England were offered incentives to refer people newly diagnosed with diabetes to structured education, and self-management programs were only eligible for accreditation if they followed a structured pathway with a clear curriculum and learning goals. In response to this, the HeLP-Diabetes researchers developed “HeLP-Diabetes: Starting Out”—an additional web-based structured education course based on the content of the original HeLP-Diabetes website. Previous research has tested this structured education course within 5 general practices in London in a small sample of patients (N=791) and found that there were problems with uptake and completion [15]. No studies to date have tested the effectiveness or cost-effectiveness of HeLP-Diabetes: Starting Out in a trial or assessed the fidelity of implementing this intervention in practice.

**Figure 1:**
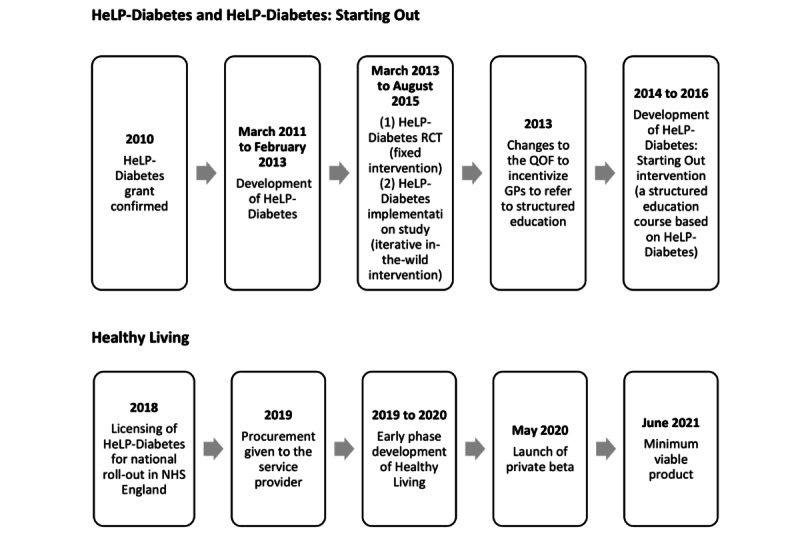
Timeline of the intervention development since 2010. GP: general practitioner; HeLP-Diabetes: Healthy Living for People With Type 2 Diabetes; NHS: National Health Service; QOF: Quality and Outcomes Framework; RCT: randomized controlled trial.

In 2019, National Health Service (NHS) England commissioned HeLP-Diabetes to be rolled out nationally in routine care under the name “Healthy Living” (Healthy Living for People With Type 2 Diabetes program). NHS England commissioned an external digital service provider to develop and offer Healthy Living as an NHS service. Figure 1 provides an overview of the development of HeLP-Diabetes, HeLP-Diabetes: Starting Out, and Healthy Living.

This study explored the fidelity of the national rollout of Healthy Living to the original HeLP-Diabetes intervention. Intervention fidelity is defined as the extent to which an intervention is delivered as intended [16]. Without good fidelity to the original HeLP-Diabetes intervention, there would be no strong justification for the implementation of Healthy Living, and reasons for intervention effectiveness would be unclear. The fidelity of diabetes self-management interventions remains largely underinvestigated [17], and fidelity evaluations are less common in routine practice than in research studies [18]. Therefore, Healthy Living presents a unique opportunity to assess the fidelity of a real-world national rollout of a digital intervention that has demonstrated effectiveness in an RCT. This study considers the extent to which Healthy Living shows fidelity to the design of the original HeLP-Diabetes intervention in relation to 3 aspects of design.

First, HeLP-Diabetes was guided by behavior change techniques (BCTs), which are the “active ingredients” of interventions that are designed to change behavior [19]. HeLP-Diabetes contained BCTs that were likely to change important health behaviors for people with T2DM, including diet, physical activity, medication adherence, alcohol consumption, and smoking. In the HeLP-Diabetes final report [20], the researchers emphasized the importance of self-regulatory BCTs, which facilitate a negative feedback loop consisting of goal setting, recognizing inconsistencies between goals and current behavior, and developing plans to mitigate these inconsistencies [21].

Second, the self-management content in HeLP-Diabetes was guided by the Corbin and Strauss [22] model for managing a long-term condition. This model states that self-management comprises 3 types of tasks: medical management (eg, adopting healthy behaviors, working with health professionals, and keeping appointments), emotional management (managing the emotions that accompany long-term conditions), and role management (changing, creating, and maintaining new meaningful life roles, such as changes in relationships, work patterns, and day-to-day activities).

Third, the effectiveness of a digital intervention is influenced by features of intervention delivery [23]. This includes all features through which the BCT and self-management content are conveyed, such as the format, materials, intensity, tailoring, and style. Therefore, it is important to assess fidelity to the features of delivery in the original HeLP-Diabetes intervention.

### Objectives

Thus, the objectives of this study were to (1) describe Healthy Living in terms of BCTs, self-management tasks, and features of intervention delivery; (2) compare the fidelity of these aspects with the original HeLP-Diabetes intervention; and (3) explain the reasons for any fidelity drift during national rollout.

## Methods

### Design

This study used a mixed methods design. A content analysis of Healthy Living was conducted using 3 coding frameworks (objective 1). The extent to which BCTs and self-management tasks were included in Healthy Living was derived and compared with published descriptions of HeLP-Diabetes (objective 2). One-to-one semistructured interviews were conducted with the key stakeholders involved in the development of HeLP-Diabetes or Healthy Living to understand the reasons for any changes during national rollout (objective 3).

### Content Analysis

#### Coding Materials

##### HeLP-Diabetes Intervention

The HeLP-Diabetes website had been deleted at the time of this study, and the intervention had not been previously coded in detail for BCTs or self-management tasks. Therefore, the following publications provided the most comprehensive description of HeLP-Diabetes: (1) the HeLP-Diabetes final report [20]; (2) a journal article describing the theoretical content of the HeLP-Diabetes intervention [24]; and (3) journal articles relating to the 3 pre-existing behavior change interventions that were integrated into the HeLP-Diabetes intervention—DownYourDrink [25,26], POWeR [27,28], and StopAdvisor [29,30].

##### Healthy Living

The content analysis assessed all aspects of the Healthy Living service available to users in June 2021. At this point, the website was in the “private beta” phase of service development, where a limited number of people with diabetes were invited to use the service and offer feedback to improve it [31]. At this stage, the website was classified as a “minimum viable product,” meaning that all the core features were in place and unlikely to change but it was still undergoing refinement [32].

Healthy Living comprised the components outlined in Textbox 1.

**Components of the Healthy Living intervention.**

- A website of 895 web pages containing written articles, videos, self-assessment quizzes, and tools; website content was broken down into 3 main components (refer to Multimedia Appendix 1 for screenshots):
  “Learn”: a structured curriculum where users worked through modules in a linear fashion, based on the Healthy Living for People With Type 2 Diabetes (HeLP-Diabetes): Starting Out website [23]
  “Find answers”: sections dedicated to various topics relating to type 2 diabetes mellitus where users could dip in and out of different pages and sections, based on the HeLP-Diabetes website [21]
  “Tools”: a range of “Goals” and “Tracker” tools based on the HeLP-Diabetes website
- Communication with users via email to encourage engagement

#### Coding Procedures

Content analysis of Healthy Living was carried out using 3 coding frameworks between June 2021 and October 2021.

##### BCT Content

The BCT Taxonomy v1 [19] defines 93 distinct BCTs and offers a reliable and valid method for coding the BCT content of behavior change interventions [33]. BCT coding was carried out independently by the first author, who underwent training in the use of the BCT Taxonomy v1 [34]. Coding was performed using data collection forms and coding procedures that have previously been used to code intervention design [35,36] (see Multimedia Appendix 2 for the BCT coding instructions and data collection checklist).

A second author (REH) double-coded 30 web pages of the Healthy Living website to assess the interrater reliability of BCT coding. These 30 web pages were purposively selected to ensure diversity in the type of web page (eg, written article, video, and quiz) and topics (eg, physical activity, working with diabetes, and emotional management).

The following health behaviors were coded as they were the target behaviors in the development of HeLP-Diabetes [20]: diet, physical activity, alcohol consumption, smoking, and medication adherence. Additional health behaviors that were identified in Healthy Living but were not the key target behaviors in HeLP-Diabetes were also coded (eg, sleep-related behaviors and sexual health behaviors). A new instance of a BCT was coded on commencement of a new activity (eg, a new web page or a video on a web page) or if a different health behavior was targeted (eg, diet or smoking). A new instance of a BCT was coded for the technique “Information about health consequences” when a different level of health behavior was targeted (eg, levels of the target behavior “diet” included information about carbohydrates, fats, and sugar). The number of distinct instances of BCTs on each web page was calculated.

##### Self-management Content

In the absence of a published coding framework for assessing self-management tasks, the authors developed a new set of coding rules. A prespecified list of self-management tasks under each of the 3 types of self-management in the Corbin and Strauss [22] model (medical, emotional, and role management) was created through team discussion (Multimedia Appendix 3). This prespecified list of self-management tasks was informed by the HeLP-Diabetes final report and additional literature on self-management in people with T2DM [37-39].

Self-management tasks were coded for each web page if at least one of the prespecified self-management tasks (Multimedia Appendix 3) was addressed. Coding was intended to assess the extent to which the intervention addressed all aspects of self-management rather than how well. Therefore, self-management tasks were coded if a task was addressed regardless of the nature of the content (eg, basic information provision, advice, and prompting of self-assessment). More than one type of self-management task could be coded on a single web page; for example, both medical and role management were coded if a web page provided information about checking blood sugar levels (medical) before driving (role).

##### Features of Intervention Delivery

Healthy Living was described using the Template for Intervention Description and Replication (TIDieR) framework for describing complex interventions [40]. TIDieR items (eg, materials, procedures, and modes of delivery) were extracted by the first author from the Healthy Living materials. The TIDieR description was member checked [41] by representatives from NHS England and the service provider for accuracy.

##### Comparison With HeLP-Diabetes

BCTs specified in the HeLP-Diabetes publications [20,24-30] were extracted into a separate data collection form for comparison with the BCTs identified in Healthy Living.

No comparison was attempted for self-management tasks as the HeLP-Diabetes publications did not provide an exhaustive description of self-management tasks to facilitate a meaningful comparison.

The TIDieR framework has previously been used to describe the HeLP-Diabetes intervention in detail [24], which enabled comparison with Healthy Living in terms of features of delivery.

#### Analysis

The Cohen κ coefficient [42] was used to assess interrater reliability between the 2 authors who independently coded 30 web pages for BCTs. κ values were calculated for each web page, and the mean of all 30 web pages was calculated. Any coding discrepancies were discussed between the authors until agreement was reached.

The presence and frequency of specific BCTs were compared between Healthy Living and HeLP-Diabetes to assess whether both used similar techniques to achieve behavior change. Particular attention was given to a comparison of the self-regulatory BCTs (eg, goal setting and action planning) as these were identified as important in the development of HeLP-Diabetes. The proportion of additional BCTs that were identified in Healthy Living but were not specified in HeLP-Diabetes was also calculated.

### Qualitative Interviews

#### Sampling and Recruitment

To ensure that the interviews provided an in-depth understanding of the reasons for any changes during national rollout, a purposive sampling strategy was used to select stakeholders who had a high level of involvement in the development of HeLP-Diabetes or Healthy Living. Stakeholders involved in the development of HeLP-Diabetes were identified by emailing members of the original academic research team. Healthy Living stakeholders were sampled through discussions with NHS England and the service provider. Additional stakeholders were identified via snowball sampling. Views were sought from stakeholders in a range of professional roles, including academic researchers, digital content developers, and program managers. As the population who could usefully comment on the intervention development process was small, the sample interviewed was small; hence, the population of interest was exhausted through the sampling strategy used.

#### Procedure and Materials

Topic guides were used to organize the semistructured interviews, with open-ended questions and additional probes. Topics covered participants’ knowledge and understanding of the HeLP-Diabetes intervention content and features of delivery, how Healthy Living had changed from the original intervention, and the reasons for any changes. All interviews were audio recorded using an encrypted audio recording device following full verbal or written consent.

#### Analysis

Audio recordings were transcribed verbatim by an external transcription company and thematically analyzed using a modified framework approach [43]. The first author read and reread transcripts, noting key ideas, and then independently coded the first 4 interviews, generating a combination of data-driven and a priori thematic codes. The data-driven codes were generated inductively from the data alone without reference to other sources. The a priori codes were based on the author’s understanding of what had changed from the original HeLP-Diabetes intervention and participants’ explicit rationale for any changes. The codes were summarized into initial themes, which were refined through discussion between 2 authors (JSB and DPF). These themes were then systematically applied to the remaining interviews, with ongoing adaptations until no new themes emerged. Themes were discussed at length between all authors until an agreement was reached on the final themes. The data were coded electronically using NVivo (version 12; QSR International).

### Ethics Approval

The wider program of research of which this study is a part was reviewed and approved by the Yorkshire and the Humber – Leeds West NHS Research Ethics Committee (reference 20/YH/0250, September 29, 2020). Full verbal or written consent was obtained from all interview participants. Interview data were anonymized at the point of transcription.

## Results

### BCT Content

#### Interrater Reliability

The mean κ value for the coding of BCTs was 0.80 (SD 0.31), thus demonstrating strong agreement [42] between coders before resolving discrepancies (see Multimedia Appendix 4 for all κ values).

#### Healthy Living

Table 1 shows the number of distinct instances of BCTs identified in Healthy Living. There were 43 BCTs identified in Healthy Living. The most common BCT was information about health consequences (849/2088, 40.7%). Diet was the behavior most commonly targeted by BCTs (targeted by 659/2088, 31.6% of all BCTs), followed by physical activity (471/2088, 22.6%) and medication adherence (454/2088, 21.7%). Multimedia Appendix 5 shows the frequency of BCTs by each health behavior.

**Table 1:** Instances of behavior change techniques (BCTs) in Healthy Living and how this compares with Healthy Living for People With Type 2 Diabetes (HeLP-Diabetes).

BCTs		Healthy Living components, n (%)					Healthy Living (all 895 pages; n=2088), n (%)		Specified in HeLP-Diabetes?
		“Learn” (273 pages; n=568)	“Find answers” (583 pages; n=1401)	“Tools” (39 pages; n=110)	Email communication (20 messages; n=9)				
**Self-regulatory BCTs**									
	Problem-solving	10 (1.8)	46 (3.3)	14 (12.7)	0 (0)	70 (3.4)		Yes	
	Self-monitoring of outcomes of behavior	17 (3)	43 (3.1)	6 (5.5)	2 (22.2)	68 (3.3)		Yes	
	Goal setting (behavior)^a^	32 (5.6)	18 (1.3)	9 (8.2)	1 (11.1)	60 (2.9)		Yes	
	Review behavior goals	17 (3)	2 (0.1)	23 (20.9)	0 (0)	42 (2)		Yes	
	Action planning	4 (0.7)	14 (1)	16 (14.5)	0 (0)	34 (1.6)		Yes	
	Self-monitoring of behavior	1 (0.2)	21 (1.5)	4 (3.6)	2 (22.2)	28 (1.3)		Yes	
	Goal setting (outcome)^a^	7 (1.2)	4 (0.3)	3 (2.7)	1 (11.1)	15 (0.7)		No	
	Feedback on behavior	9 (1.6)	3 (0.2)	2 (1.8)	0 (0)	14 (0.7)		Yes	
	Biofeedback^a^	4 (0.7)	9 (0.6)	0 (0)	0 (0)	13 (0.6)		No	
	Review outcome goals	3 (0.5)	2 (0.1)	5 (4.5)	1 (11.1)	11 (0.5)		No	
	Feedback on outcomes of behavior	0 (0)	0 (0)	3 (2.7)	0 (0)	3 (0.1)		Yes	
	Commitment	0 (0)	0 (0)	0 (0)	0 (0)	0 (0)		Yes	
**Other BCTs**									
	Information about health consequences	232 (40.8)	617 (44)	0 (0)	0 (0)	849 (40.7)		Yes	
	Social support (unspecified)	73 (12.9)	133 (9.5)	2 (1.8)	2 (22.2)	210 (10.1)		Yes	
	Information about emotional consequences	41 (7.2)	68 (4.9)	0 (0)	0 (0)	109 (5.2)		Yes	
	Behavior substitution	33 (5.8)	57 (4.1)	8 (7.3)	0 (0)	98 (4.7)		No	
	Credible source	22 (3.9)	21 (1.5)	0 (0)	0 (0)	43 (2.1)		No	
	Instruction on how to perform the behavior	0 (0)	41 (2.9)	0 (0)	0 (0)	41 (2)		Yes	
	Social support (practical)	12 (2.1)	29 (2.1)	0 (0)	0 (0)	41 (2)		No	
	Information about social and environmental consequences	8 (1.4)	31 (2.2)	0 (0)	0 (0)	39 (1.9)		No	
	Information about antecedents	2 (0.4)	36 (2.6)	0 (0)	0 (0)	38 (1.8)		Yes	
	Behavioral practice or rehearsal	4 (0.7)	32 (2.3)	0 (0)	0 (0)	36 (1.7)		Yes	
	Demonstration of the behavior	3 (0.5)	32 (2.3)	0 (0)	0 (0)	35 (1.7)		Yes	
	Adding objects to the environment	4 (0.7)	24 (1.7)	4 (3.6)	0 (0)	32 (1.5)		Yes	
	Reduce negative emotions	5 (0.9)	16 (1.1)	0 (0)	0 (0)	21 (1)		Yes	
	Restructuring the physical environment	6 (1.1)	13 (0.9)	2 (1.8)	0 (0)	21 (1)		Yes	
	Restructuring the social environment	2 (0.4)	13 (0.9)	4 (3.6)	0 (0)	19 (0.9)		No	
	Social support (emotional)	4 (0.7)	13 (0.9)	0 (0)	0 (0)	17 (0.8)		No	
	Prompts and cues	3 (0.5)	10 (0.7)	2 (1.8)	0 (0)	15 (0.7)		Yes	
	Increase positive emotions^b^	2 (0.4)	7 (0.5)	0 (0)	0 (0)	9 (0.4)		No	
	Nonspecific reward	0 (0)	9 (0.6)	0 (0)	0 (0)	9 (0.4)		No	
	Self-reward	0 (0)	9 (0.6)	0 (0)	0 (0)	9 (0.4)		Yes	
	Avoidance or reducing exposure to cues for the behavior	0 (0)	5 (0.4)	2 (1.8)	0 (0)	7 (0.3)		Yes	
	Distraction	0 (0)	7 (0.5)	0 (0)	0 (0)	7 (0.3)		No	
	Salience of consequences	5 (0.9)	2 (0.1)	0 (0)	0 (0)	7 (0.3)		No	
	Pros and cons	2 (0.4)	3 (0.2)	0 (0)	0 (0)	5 (0.2)		Yes	
	Pharmacological support	0 (0)	3 (0.2)	0 (0)	0 (0)	3 (0.1)		Yes	
	Material incentive (behavior)	0 (0)	2 (0.1)	0 (0)	0 (0)	2 (0.1)		No	
	Self-incentive	0 (0)	2 (0.1)	0 (0)	0 (0)	2 (0.1)		No	
	Social incentive	0 (0)	2 (0.1)	0 (0)	0 (0)	2 (0.1)		No	
	Mental rehearsal of successful performance	0 (0)	1 (0.1)	0 (0)	0 (0)	1 (0)		No	
	Reattribution	0 (0)	1 (0.1)	0 (0)	0 (0)	1 (0)		No	
	Salience of behavior^c^	0 (0)	0 (0)	1 (0.9)	0 (0)	1 (0)		No	
	Social reward	1 (0.2)	0 (0)	0 (0)	0 (0)	1 (0)		Yes	
	Conserving mental resources	0 (0)	0 (0)	0 (0)	0 (0)	0 (0)		Yes	
	Graded tasks	0 (0)	0 (0)	0 (0)	0 (0)	0 (0)		Yes	
	Identity associated with changed behavior	0 (0)	0 (0)	0 (0)	0 (0)	0 (0)		Yes	
	Identification of self as role model	0 (0)	0 (0)	0 (0)	0 (0)	0 (0)		Yes	
	Information about others’ approval	0 (0)	0 (0)	0 (0)	0 (0)	0 (0)		Yes	
	Self-talk	0 (0)	0 (0)	0 (0)	0 (0)	0 (0)		Yes	
	Social comparison	0 (0)	0 (0)	0 (0)	0 (0)	0 (0)		Yes	

^a^Includes BCTs that were “prompted” rather than directly delivered regardless of whether the BCT Taxonomy v1 (BCTTv1) definition specified that the BCT can be prompted. For example, the definition in the BCTTv1 for goal setting (behavior) was as follows: “Set or agree on a goal defined in terms of the behavior to be achieved.” However, this BCT was coded when patients were prompted to set a goal elsewhere as part of the intervention (eg, by clicking on the “Physical activity goal” tool). Refer to Multimedia Appendix 3 for further details on BCT coding procedures.

^b^Increase positive emotions is not listed in the BCTTv1 but was noted by the authors for inclusion in the next version of the taxonomy.

^c^Salience of behaviors is not listed in the BCTTv1 but has been identified as a new BCT by the authors of this paper.

#### Comparison With HeLP-Diabetes

There were 32 BCTs specified in the HeLP-Diabetes intervention (Table 1). Healthy Living included 75% (24/32) of these BCTs (Table 1). There were an additional 37% (19/51) of BCTs that were identified in Healthy Living but were not specified in HeLP-Diabetes (Table 1).

All but one of the self-regulatory BCTs (“commitment”) specified in HeLP-Diabetes were identified in Healthy Living, including problem-solving (70/2088, 3.4%), self-monitoring of outcomes of behavior (68/2088, 3.3%), self-monitoring of behavior (28/2088, 1.3%), goal setting (for behavior; 60/2088, 2.9%), review behavior goals (42/2088, 2%), action planning (34/2088, 1.6%), feedback on behavior (14/2088, 0.7%), and feedback on outcomes of behavior (3/2088, 0.1%; Table 1). There were also other self-regulatory BCTs identified in Healthy Living that were not explicitly specified in the HeLP-Diabetes final report, including goal setting (for outcomes; 15/2088, 0.7%), biofeedback (13/2088, 0.6%), and review outcome goals (11/2088, 0.5%; Table 1).

### Self-management Content

The number of distinct instances of self-management tasks that were addressed in Healthy Living is summarized in Table 2. Most of the Healthy Living intervention addressed medical management tasks (821/895, 91.7% of all web pages). Emotional management tasks were addressed in 35.4% (317/895) of all web pages, and role management tasks were addressed in 30.9% (277/895) of all web pages.

**Table 2:** Instances of self-management tasks addressed in Healthy Living.

Self-management tasks	Healthy Living components, n (%)				Healthy Living (all 895 pages; n=1415), n (%)
	“Learn” (273 pages; n=392)	“Find answers” (583 pages; n=975)	“Tools” (39 pages; n=41)	Email communication (20 messages; n=7)	
Medical	211 (53.8)	565 (57.9)	38 (92.7)	7 (100)	821 (58)
Emotional	103 (26.3)	211 (21.6)	3 (7.3)	0 (0)	317 (22.4)
Role	78 (19.9)	199 (20.4)	0 (0)	0 (0)	277 (19.6)

### Features of Intervention Delivery

#### Healthy Living

Multimedia Appendix 6 [14,15,40,44-51] contains a detailed description of Healthy Living using the TIDieR framework. In brief, Healthy Living was a free digital NHS service for people living with T2DM developed for use on a range of digital devices (ie, smartphones, desktops, and tablets). The website contained 895 web pages, including information about what T2DM is, its causes, and how it can be managed and treated; behavioral advice on diet, physical activity, alcohol, smoking, and medication adherence; and emotional and practical support. There were tools for users to set and review goals, make action plans, and self-monitor. Healthy Living was intended for people diagnosed with T2DM in England, carers, and health care professionals. The service was available by self-referral, although there were ongoing plans to develop primary care referral once beta testing was complete and once health services opened up after the COVID-19 pandemic. Technical support was provided, but there were no health care professionals to support the use of the website or behavior change.

#### Comparison With HeLP-Diabetes

When comparing Healthy Living with HeLP-Diabetes, there were similarities in relation to the written content and topics (eg, understanding diabetes and preexisting interventions) and types of materials (eg, articles, videos, and tools).

However, there were a number of important differences (summarized in Table 3). The HeLP-Diabetes intervention offered patients facilitated access through a 5- to 10-minute onboarding process with a health care professional in primary care. There were ongoing plans to develop primary care and community hub referral pathways for Healthy Living, but there were no plans to include facilitated access by a health care professional. The Healthy Living website also included an additional structured curriculum, which was based on the HeLP-Diabetes: Starting Out course developed after the RCT by the HeLP-Diabetes team [15]. However, this structured curriculum was not included in the original HeLP-Diabetes intervention tested in the RCT. Features of the HeLP-Diabetes website that were not retained in Healthy Living included a moderated forum (where users could interact with other users and ask health professionals questions) and a health record (where users could record and keep track of appointments and tests with health care professionals).

**Table 3:** Summary of differences in the features of delivery between Healthy Living for People With Type 2 Diabetes (HeLP-Diabetes), HeLP-Diabetes: Starting Out, and Healthy Living.

Feature	“HeLP-Diabetes” (RCT^a^ version)	“HeLP-Diabetes: Starting Out”	“Healthy Living”
Registration	Facilitated access by a practice nurse through a 5- to 10-minute appointment	Self–sign-up, with optional telephone support for those who had difficulty registering or using the website	Self–sign-up (plans to develop referrals through primary care and community hubs but no facilitated access)
Size of website	8 sections, 560 pages	5 sections, with selected content from HeLP-Diabetes; users also had access to the HeLP-Diabetes website via a common home page	3 sections, 895 pages
How the intervention was delivered	Nonlinear—users could access any part of the website and dip in and out as they pleased in any order	Linear and nonlinear—users worked through modules one by one but also had access to the nonlinear component, where they could dip in and out of sections as they pleased in any order	Linear and nonlinear—users worked through modules one by one but also had access to the nonlinear component, where they could dip in and out of sections as they pleased in any order
Curriculum	No curriculum—users could choose which topics to access depending on interest	Structured curriculum—users had access to a series of modules that could be worked through in a spiral fashion	Structured curriculum—users had access to a series of modules that could be worked through in a spiral fashion
Forum and help	There was a moderated web-based forum where users could interact with other users, and there was an “Ask the Expert” option where users could ask health professionals questions; additional resources included local resources tailored to the CCG^b^ and a list of frequently asked questions	Users had access to the HeLP-Diabetes nonlinear website, where they could access the web-based forum, “Ask the Expert,” and all the additional tailored resources	No moderated web-based forum or tailored support
Health record	Users could record and keep track of appointments with health care professionals and of the results of tests used to monitor diabetes (eg, HbA1c, blood pressure, cholesterol level, and kidney and liver function)	Users had access to the HeLP-Diabetes website, where they could record and keep track of health care appointments and test results	No health record, but an HbA1c tracker was offered
Physical materials	Practice nurses were provided with training leaflets for facilitated access; information leaflets for patients	No physical materials were offered	Information leaflets for patients in primary care
Engagement	Email, SMS text message reminders, and follow-up phone calls	Emails only	Emails only

^a^RCT: randomized controlled trial.

^b^CCG: clinical commissioning group.

### Qualitative Interviews

#### Participants

A total of 9 participants were interviewed, including stakeholders from the HeLP-Diabetes academic team (n=5, 56%), the NHS England diabetes program team (n=1, 11%), and the service provider (n=3, 33%). Most interviews (8/9, 89%) were carried out between May 2021 and June 2021, and 11% (1/9) of the interviews were conducted in September 2020. Interviews lasted between 46 and 102 (mean 70) minutes.

A total of 3 overarching themes were identified: changes because of scalability issues, changes to improve user engagement and outcomes, and digital development challenges.

#### Theme 1: Scalability Issues

The NHS England interviewee indicated that some features from the original HeLP-Diabetes intervention were not implemented as there were fewer resources (staff and time) within general practices compared with the RCT to deliver these features at scale. Issues of scalability predominantly related to features that required additional support from health care professionals.

##### Subtheme 1.1: Fewer Resources Available to Provide Facilitated Access

The NHS England interviewee reported that health care professionals would be unable to spend time supporting patients to register and onboard them to the intervention because of capacity issues and time constraints that were not necessarily an issue in the RCT. Interviewees from the HeLP-Diabetes team said that they encountered similar difficulties in their implementation research, which was conducted in parallel to the RCT:

We already felt that it wasn’t scalable and that we were already very aware that it would have been a big request of general practices to spend time onboarding the user, when we were already aware of some of the capacity issues and time constraints within an annual review already...We worked with the early engagement areas to sort of validate that understanding and it was a universal confirmation that they would not implement if we maintained that onboarding mechanism.

Participant 4, NHS England

When asked about the implications of removing facilitated access to the intervention, the NHS England interviewee believed that it would not necessarily have a negative impact on patients as the program would still involve referral from a health care professional, thus retaining the trust associated with health care professional recommendations. This interviewee also believed that removing facilitated access increased buy-in from health care professionals, which was important to encourage patient referrals to the intervention.

Although acknowledging implementation issues, interviewees from the HeLP-Diabetes team suggested that facilitated access was an important component of the original intervention to support people with lower education or limited computer skills:

Our ideal model was facilitated access. Someone from the surgery, it didn’t have to a doctor, but maybe a nurse or someone like that could sign you up thereby, signposting that this was a recommended intervention, but also help overcome any initial inertia of a digital device, showing people around, this is how you can log on, this is your password.

Participant 2, HeLP-Diabetes

##### Subtheme 1.2: Omission of the Moderated Web-Based Forum

The service provider was informed by the HeLP-Diabetes team that the uptake of the forum was quite low in the RCT, in part because the RCT did not “reach the critical mass of users to populate the forum” (participant 1, HeLP-Diabetes). As a result, participants generally reported that the moderated forum was not perceived to be an integral feature of the intervention. Therefore, the service provider preferred not to invest substantial staff time in the moderation of a web-based forum, which had been underused in the RCT, and this was supported by NHS England:

The decision was made to drop that particular feature of the programme [the moderated forum]. So, it was obviously partly because we had heard from the HeLP team that the uptake had not been great, and then there was also not the resource to support it in the way it would need.

Participant 9, service provider

##### Subtheme 1.3: Encouraging Users to Seek External Support

The moderated forum in the original HeLP-Diabetes website included an “Ask the expert” option, where users could submit questions to a health care professional as “a way of getting help and advice” (participant 3, HeLP-Diabetes). However, the interviewee from NHS England believed that this was not a scalable option for a national program because of limited resources. Furthermore, providing clinical advice would require access to patient medical records and, without this, the service provider would only be able to provide generic advice:

[‘Ask the expert’] wasn’t a scalable option for the programme...There’s information for a user to self-manage, but there is clear direction throughout the programme that if they need something specific for their self-management, that they need to speak to their own healthcare professional.

Participant 4, NHS England

#### Theme 2: Improving User Engagement and Outcomes

The service provider believed that the original HeLP-Diabetes website needed modifying to improve engagement and outcomes for users.

##### Subtheme 2.1: Perceived Importance of the Structured Curriculum

On the basis of their experiences from other projects, the service provider believed that the structured curriculum (“Learn”) provided the most effective way of improving patient outcomes and monitoring patients’ progress. As a result, they perceived the structured curriculum “as the core content” (participant 7, service provider) and wanted most patients to use the structured curriculum, although they acknowledged that some users would prefer to engage in free exploration that was offered in the “Find answers” component of Healthy Living:

We are quite geared towards encouraging people to the Learn Journey because it is a structured programme, it quite often will get better results. Like we’ve seen in other programmes, if someone completes a certain percentage of a learning programme, they’re more likely to achieve the outcomes and the goals that they’re setting alongside it.

Participant 9, service provider

When originally designing the intervention, interviewees from the HeLP-Diabetes team expressed difficulties in grappling with the decision of whether to include a structured curriculum. Even though the evidence base suggested that a structured curriculum was more likely to be effective, participants’ main concern was that it was difficult to get users to complete an entire curriculum, especially without additional support or encouragement. Interviewees from the HeLP-Diabetes team explained that patients in their qualitative research said that they would prefer to have access to self-management information as and when they needed it rather than having the burden of completing modules in a prescribed manner; this was a key factor in not including a structured curriculum in the original HeLP-Diabetes intervention:

There was definitely a big debate in it, because I think in the literature there was some evidence to say if it’s structured it’s more likely to be effective, but in all of our qualitative work, people didn’t want it like that. But I know we did have a bit of back and forth, but we were mainly led from our work with people that were going to use it, who just sort of said that that would really put them off using it full stop, if it was that sort of more structured, and that they felt that they just wanted to come and be able to dip in and dip out, search for things, and use bits from websites that they felt they needed in that moment.

Participant 3, HeLP-Diabetes

##### Subtheme 2.2: User Research

An independent user research organization was commissioned by NHS England to conduct user research to inform the development of Healthy Living. This was required as part of the UK Government Digital Service Standard [44] that was originally published in 2016. This user research was perceived as having a positive impact on user engagement, especially as the original HeLP-Diabetes website was perceived as “overwhelming” (participant 4, NHS England) and “visually cluttered” (participant 9, service provider). Interviewees from the service provider reported examples in which this user research led to modifications of the original HeLP-Diabetes website, often because of changes in how people now use digital technology:

The medication tracker was not a particularly popular one [from user research]. I think, if I remember rightly, I think in the time that the HeLP programme was live, people have become a lot more dependent on a mobile phone for their reminders and prompts and things like that, and the notion of using a third-party website to support that was perhaps a bit less attractive to people by that point.

Participant 9, service provider

##### Subtheme 2.3: Data-Driven Optimization of the Intervention

Participants recognized the potential of using data analytics to assess and iteratively modify the website to improve user engagement over time. The service provider described how they planned to continually use real-time data to improve the website as they did not perceive the current website as the definitive version:

You also have real time analytics, so that you can then start to evolve that programme in real time to optimise retention, completion outcomes.

Participant 6, service provider

#### Theme 3: Digital Development Challenges

The service provider experienced a number of digital challenges when developing the original website.

##### Subtheme 3.1: Adhering to Digital Standards

The service provider emphasized the challenges of adhering to digital standards, including the UK Government Digital Service Standard [44], Web Content Accessibility Guidelines [45], Digital Technology Assessment Criteria [46], and NHS Digital content [47] and style guidelines [48]. The service provider had to reformat a lot of the original content to abide by these standards, which included “making the content more readable for the average reader” (participant 4, NHS England), “reducing some of the repetition” (participant 9, service provider), and “reformatting the way that the information is provided so that it is in smaller chunks” (participant 4, NHS England). This process of adapting the content to meet digital standards was a new experience for the service provider and was perceived as arduous, especially given the sheer amount of content on the original website. Despite these challenges, interviewees from the service provider were pleased with the end result:

We’ve certainly had quite a steep learning curve around the GDS [Government Digital Service] assessment process that sits alongside the work, that was kind of a new area for us, and I think for many of the NHS team as well...I would say we’ve learned an awful lot in the process of this project around GDS in itself, but I think we’re at a point now where what we have is a good product.

Participant 9, service provider

##### Subtheme 3.2: Lack of Iterative Development Over Time

The service provider said that the HeLP-Diabetes website had not undergone any iterative development for a number of years, which was an issue owing to the significant level of technological evolution in the time since the original website was developed. The service provider felt that they had to significantly update the website in line with current user expectations of a digital service. Interviewees from the HeLP-Diabetes team acknowledged that they had limited internal web development expertise, which was problematic for developing the website and keeping it up-to-date with advances in technology:

As a research project that’s fundamentally not based in tech, an outside company came in and did the tech for us. But I think that was a real limitation in terms of keeping up to date with the tech and helping the intervention adapt change to the different ways in which people engage with physical content.

Participant 2, HeLP-Diabetes

##### Subtheme 3.3: Reverse Engineering the Original Website

The service provider explained that they did not receive any documents to help them build the original website, such as a site map or a master file of content, which is what would usually happen in other similar digital projects that they had worked on. This meant that they were forced to spend a lot of time working out what the original content was and how it was structured to “reverse engineer the website” (participant 6, service provider). An interviewee from the service provider highlighted the difficulties of translating the underlying theory and BCTs into a digital service:

People talk a lot about behavioural frameworks and behaviour change techniques. And then, they talk very little about how they’ve operationalised them in a digital service. And I think there’s a big gap there, because it’s easy to write a behavioural framework, it’s hard to show how it works in the digital intervention.

Participant 6, service provider

## Discussion

### Summary of Principal Findings

The national rollout of Healthy Living included all but one (“commitment”) of the self-regulatory BCTs that were specified in the original HeLP-Diabetes intervention, including goal setting, self-monitoring, and problem-solving. Healthy Living predominantly addressed medical self-management tasks (821/895, 91.7% of web pages) but also addressed emotional (317/895, 35.4% of web pages) and role (277/895, 30.9% of web pages) self-management tasks. Therefore, the national rollout of Healthy Living had good fidelity to the BCT and self-management content of HeLP-Diabetes.

However, there were a number of changes to features of delivery during the national rollout, two of which are most noteworthy. First, the Healthy Living service included an additional structured learning curriculum that was developed after the RCT by the HeLP-Diabetes team but was not part of the HeLP-Diabetes intervention tested in the RCT. Second, Healthy Living did not implement features that required health care professional support as NHS England believed that they were not scalable. The interviewees described how the service provider had to substantially reformat the content of the original HeLP-Diabetes website to make it more usable and accessible, which was a requirement to meet digital standards to allow the intervention to be scaled up for national implementation in the NHS.

### Strengths and Limitations

This fidelity analysis used 3 coding frameworks to assess every page of the Healthy Living website and all email communications offered to users, thus providing a comprehensive fidelity assessment. The authors developed a bespoke framework to assess self-management tasks as existing taxonomies were insufficient to code other aspects of interventions beyond behavior change; to the authors’ knowledge, this study is the first to quantify the self-management content of an intervention. Further work is needed to develop this self-management coding framework to ensure applicability for a broad range of interventions and chronic conditions and validate the framework. Although the qualitative sample was small, there were only a limited number of stakeholders with a high level of involvement in the intervention development process (ie, we included all the population of interest). A further strength is that this study was conducted independently of those involved in the development of the intervention, which is rare in previous fidelity assessments [52].

Nevertheless, there are limitations to consider. This study was conducted at a relatively early phase in the development of Healthy Living, so there may be further changes to the website that would potentially alter these findings. This is a common challenge when assessing the fidelity of digital interventions because of the fast-moving pace of digital technology [53]. This evaluation was also conducted before the national implementation of primary care referral pathways into Healthy Living, which was delayed because of disruptions caused by the COVID-19 pandemic. However, as of September 2021, NHS England and the service provider had no plans to provide facilitated access or make major changes to the website content, so it is unlikely that conducting this evaluation when primary care referrals are implemented will alter the conclusions. Finally, the authors could not precisely compare the number of instances of BCTs and self-management tasks between Healthy Living and HeLP-Diabetes as the original HeLP-Diabetes website was not available to us, having been discontinued at the time of evaluation. Not having access to the original intervention website is an example of one of the many challenges of a fidelity evaluation conducted by a research team that is independent of those who developed the original intervention. Instead, we relied on published papers relating to HeLP-Diabetes, which described the BCT and self-management task content in great detail but did not specify where on the website they occurred or how often.

### Comparison With Prior Work

To the authors’ knowledge, this is the first in-depth fidelity assessment of a nationally implemented digital diabetes self-management intervention. The findings reported in this paper are in line with research assessing the fidelity of design of the NHS Digital Diabetes Prevention Programme (NHS-DDPP), a behavioral intervention for people identified as at high risk of developing T2DM. The researchers found that 85% of the BCTs outlined in the NHS-DDPP specification (which emphasized the importance of self-regulatory BCTs) were included in the service providers’ intervention plans [54]. In contrast, a similar evaluation of the face-to-face version of the NHS Diabetes Prevention Program found that only 37% of specified BCTs were delivered during the program [55], and some core self-regulatory BCTs were underdelivered in the observed sessions, such as goal setting, which was delivered in 52.5% of sessions [56]. Healthy Living compares favorably with these other interventions, with 75% (24/32) of the BCTs specified in HeLP-Diabetes identified in Healthy Living, including all but one of the self-regulatory BCTs (Table 1). An explanation for the 25% (8/32) of BCTs missing in Healthy Living may be that information on the BCT content was distributed across multiple documents and was not collated for the service provider; this contrasts with the NHS Diabetes Prevention Programme and NHS-DDPP, where there were prespecified lists of BCTs that were stipulated to be delivered. The high fidelity of BCTs in digital interventions compared with face-to-face interventions is in line with the literature, which suggests that digital interventions may achieve higher fidelity as they do not rely on human delivery [57].

There were 46% (43/93) of possible BCTs offered in the Healthy Living service, which is high compared with other digital self-management interventions. For instance, a systematic review of 8 digital self-management interventions for people with T2DM found that the highest number of BCTs in an intervention was 14 [58]. This is important given that digital behavior change interventions that use more BCTs have been found to have larger effect sizes compared with interventions that use fewer BCTs [59]. Healthy Living also compared favorably with most consumer-facing smartphone apps, which have been found to implement a very limited number of BCTs [60] and often lack firm grounding in theory or evidence [61].

It is important that Healthy Living addressed all aspects of self-management, including emotional management, as previous intervention research suggests that addressing the emotional and psychological aspects of T2DM can reduce diabetes distress and improve HbA1c levels [62-65]. Previous digital interventions for people with T2DM have predominantly focused on providing information and behavior change support but less so on emotional self-management [66-69]. Hence, there have been calls for more emotional and psychological support to be embedded within routine diabetes care [70,71].

### Implications for Healthy Living

Facilitated access was not implemented in Healthy Living as there were fewer resources within the NHS than in the RCT to provide this support on a national scale. However, the HeLP-Diabetes team believed that facilitated access was important to encourage uptake, especially for those who have lower levels of education. This belief may stem from the HeLP-Diabetes implementation research, which found that “the self-sign-up model was associated with users who were better educated and had rated their computer skills as advanced” [20] when compared with the version of HeLP-Diabetes that retained facilitated access. Additional resources may need to be allocated to those Healthy Living users who might benefit from extra assistance to sign up or use the service to ensure that the intervention does not inadvertently contribute to a widening of health inequalities.

NHS policy recommends that all diabetes self-management education interventions contain a structured curriculum with clear learning objectives, which meant that a structured learning curriculum was required for national implementation. However, the stakeholder interviews in this study suggested that there is ambivalence as to whether having a structured curriculum would be more effective than a website with no structured curriculum. The evidence generally indicates that structured education interventions are effective but only if patients sufficiently engage with the intervention [72], something that is difficult to achieve for diabetes self-management education [15]. Given the low uptake previously observed in the HeLP-Diabetes: Starting Out intervention [15], improving engagement with Healthy Living will be critical for intervention effectiveness as the frequency and intensity of digital intervention use are thought to be important in achieving desired outcomes [72].

### Implications for Practice

The important changes from the original HeLP-Diabetes intervention were associated with the implementation challenges of going from an RCT to a scaled-up national program. The service specification from NHS England indicated that the service provider should aim to retain fidelity to the original intervention approach that has been evidenced in the HeLP-Diabetes RCT. Although expecting perfect fidelity is unrealistic when moving from controlled to real-world settings [73], there were 2 main problems associated with this approach of aiming to retain fidelity to the intervention from the RCT for the national rollout of Healthy Living.

First, the original RCT had significant dedicated resources that enabled a more intensive intervention through the use of dedicated health care professional support, for example, through facilitated access to support the user registration process. It was clear from the interviews that providing this level of intensity was not feasible in a scaled-up national program. The HeLP-Diabetes team originally identified this as a potential issue during their implementation research but, as this research was conducted in parallel to the RCT, it did not inform the intervention design in the RCT.

The second problem was the sheer length of time from when the HeLP-Diabetes intervention underwent testing in a trial in 2013 to the subsequent procurement of Healthy Living in 2019. This meant that, by the time the website from the RCT was due to be rolled out, it required adaptation to be consistent with the new clinical and technological environment in which it was being implemented, including changes in policy, advances in digital technology, and new standards for providing digital services.

This highlights the importance of considering implementation challenges at earlier phases of intervention development to reduce the level of adaptation necessary for scaling up an intervention to real-world contexts (often called a “scale-up penalty” [73]). Addressing this issue is likely to require a shift in the way that academic health research is funded as funding is often focused on commissioning research on effectiveness rather than on the implementation stages of digital development, both of which are necessary to create effective digital interventions that are implementable outside the context of clinical trials [74]. Health funding needs to accommodate faster and more efficient methods of evaluation that enable the iterative development of digital interventions across their life cycles [75,76], such as the Multiphase Optimization Strategy [77]. There appears to be growing recognition of this. For example, the National Institute of Health and Care Excellence recently published an Evidence Standards Framework for Digital Health Technologies to develop standards that ensure that new digital technologies are not just clinically effective and cost-effective but also enable a more dynamic approach to digital development and delivery [78]. As innovation in digital technology becomes increasingly rapid and as the NHS becomes increasingly digital, a more flexible approach to the way research is funded and conducted will become even more important.

This study highlighted the importance of multidisciplinary teams during the intervention development process and throughout implementation. Although HeLP-Diabetes had multidisciplinary working groups of service users, clinicians, and researchers, the internal HeLP-Diabetes academic team did not have the professional digital development and design knowledge required to iteratively develop a high-quality product that met evolving user expectations and digital standards. Similarly, during national implementation, the service provider reported difficulties in translating the underlying theoretical models into a digital program. Effective operationalization of BCTs to a digital context is currently underdeveloped within academic research [79], so it is unsurprising that the service provider experienced difficulties in translating BCTs into digital content. Addressing the barriers to multisectoral collaborations in digital health intervention research is necessary to ensure that expertise in health and digital software development is integrated across all stages of development, evaluation, and implementation of digital health interventions [74].

### Future Research

The authors of this study are conducting other streams of work in relation to the fidelity of the Healthy Living service, which will involve analyzing use data to assess how much of the intervention content is actually engaged with by users, as well as interviews with Healthy Living users to explore how the intervention is understood and experienced. This future research will help address some of the uncertainties identified in this study, such as the impacts of the 2 important changes in Healthy Living (lack of facilitated access and inclusion of structured education) on user engagement and experiences. This future research will also look at the extent to which Healthy Living users need tailored self-management support to help their own individual needs given that evidence suggests that self-management needs to be orientated to a person’s individual needs [80].

### Conclusions

This mixed methods study found that the national rollout of Healthy Living had good fidelity to the BCT and self-management content of HeLP-Diabetes. However, this study identified important changes that were attributable to the challenges of scaling up a digital intervention from an RCT to a nationally implemented intervention, mainly because of fewer resources available in practice and the length of time since the RCT. This study demonstrates the importance of considering implementation throughout all phases of intervention development and testing to reduce the level of adaptation necessary for scaling up an intervention to real-world contexts. Greater collaboration between academic researchers and digital development experts is needed to produce evidence- and theory-informed digital health interventions that are usable and accessible enough to meet digital standards.

## Appendix

Multimedia Appendix 1 Screenshots of the 3 main components of the Healthy Living website.

Multimedia Appendix 2 Procedures for behavior change technique coding.

Multimedia Appendix 3 Prespecified list of self-management tasks.

Multimedia Appendix 4 The κ values for assessing the interrater reliability of behavior change technique coding.

Multimedia Appendix 5 Instances of behavior change techniques in Healthy Living for each health behavior.

Multimedia Appendix 6 Description of Healthy Living using the Template for Intervention Description and Replication framework.

## Abbreviations

BCT: behavior change technique
HeLP-Diabetes: Healthy Living for People With Type 2 Diabetes
NHS: National Health Service
NHS-DDPP: National Health Service Digital Diabetes Prevention Programme
RCT: randomized controlled trial
T2DM: type 2 diabetes mellitus
TIDieR: Template for Intervention Description and Replication

## Data Availability

Some data sets are available from the corresponding author upon reasonable request, although those making the request will need to obtain the explicit permission of the relevant provider organizations.
