# Supplementary material for: Changes in a Digital Type 2 Diabetes Self-management Intervention During National Rollout: Mixed Methods Study of Fidelity
Source: J Med Internet Res. 2022 Dec 7;24(12):e39483. doi: 10.2196/39483 (PMC9773035; doi:10.2196/39483)

**Appendix 1.** Screenshots of the three main components in the Healthy Living website: ‘Learn’ (top), ‘Find answers’ (middle) and ‘Tools’ (bottom).


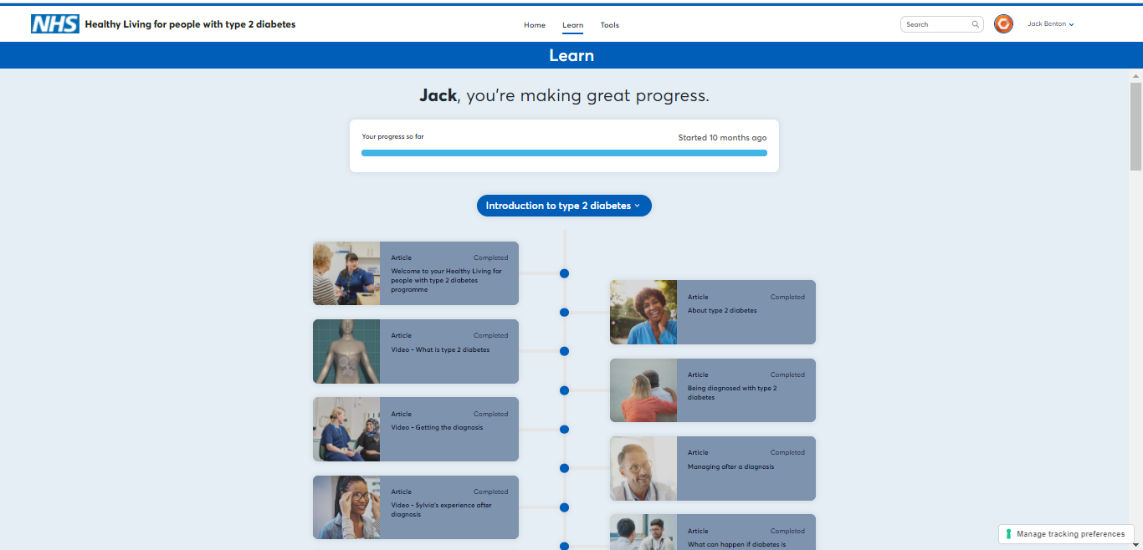


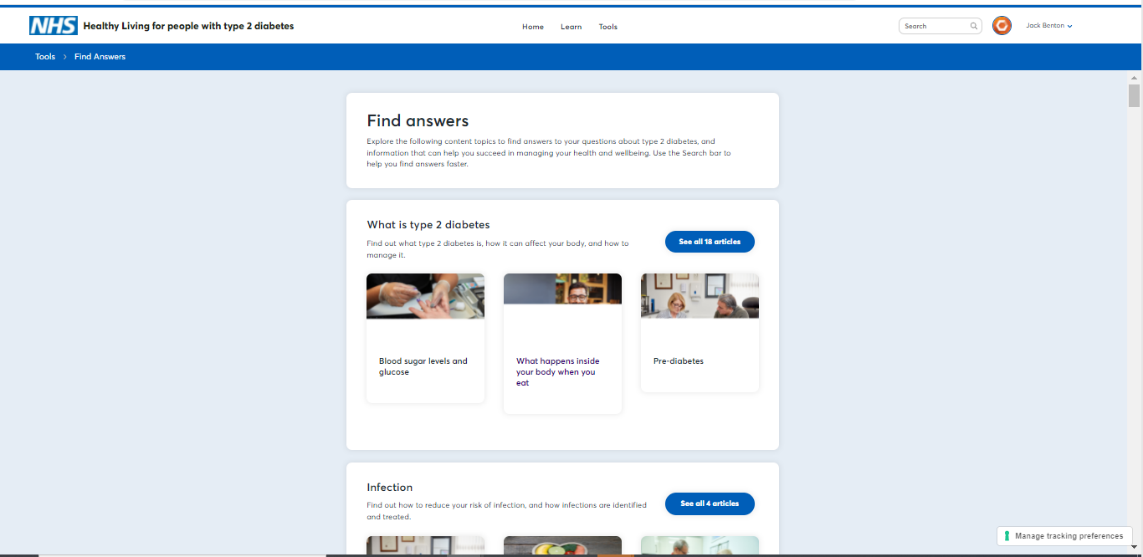


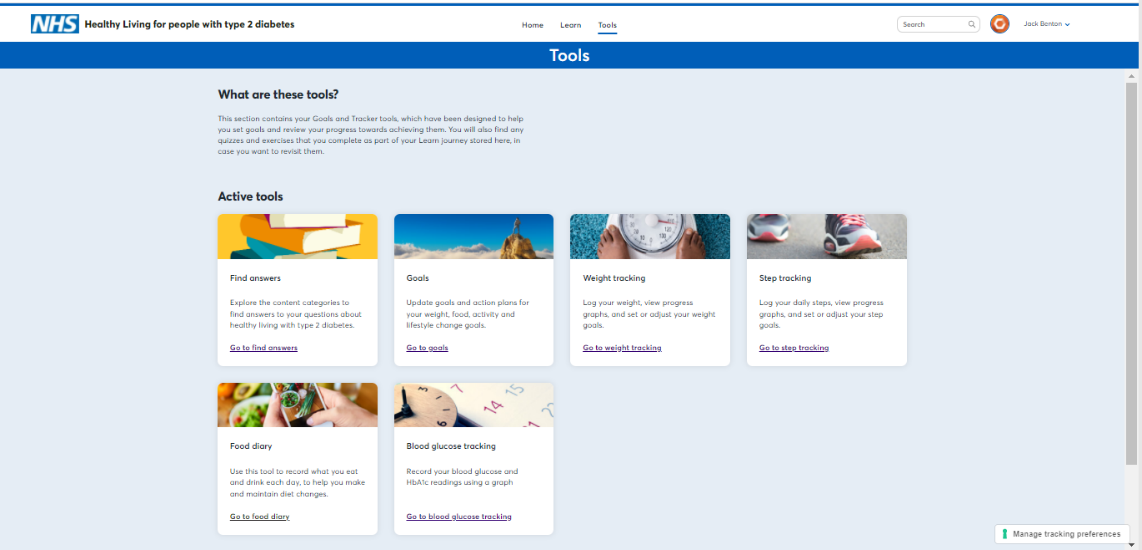

Supplement: Multimedia Appendix 1 [file jmir_v24i12e39483_app1.docx]
