# Supplementary material for: Changes in a Digital Type 2 Diabetes Self-management Intervention During National Rollout: Mixed Methods Study of Fidelity
Source: J Med Internet Res. 2022 Dec 7;24(12):e39483. doi: 10.2196/39483 (PMC9773035; doi:10.2196/39483)
Supplement: Multimedia Appendix 2 [file jmir_v24i12e39483_app2.docx]

**Appendix 3.** Procedures for BCT coding

1. List behaviour change techniques in the order they appear in the website.
2. Separate different webpages using empty rows shaded grey.
3. Code each instance where a new BCT is ***offered*** in the webpage [*Note: users may not engage with/take-up all BCTs presented*].
4. Code as a BCT if a specific BCT is mentioned (e.g. “use problem solving…”) even if the BCT is not fully explained, unless there is evidence to suggest that the BCT is not present (i.e. the explanation that is provided does not describe the BCT definition).
5. When coding BCTs, take into account the *overall context*, i.e. don’t just code the webpage in isolation:

- E.g. If users are reviewing their goals on a webpage, and we know that they set a behaviour goal on the previous webpage, we know to code ‘Review of behaviour goal(s).’

1. Keep notes on any queries or problems with the coding process. Always document *why* a coding decision was made and these can be discussed later.
2. **BCT code:** Insert code number from BCT taxonomy v1 (e.g. 2.1, 3.2).

- ‘Increase positive emotions’ is not listed in the BCTTv1, but was noted by the authors for inclusion in the next version of the taxonomy: “Advise on ways of increasing positive emotions to facilitate performance of the behaviour”. For ‘Increase positive emotions’ put the full label in the BCT label column and leave the code column blank.

1. **BCT label:** Insert label from BCT taxonomy v1 (e.g. goal setting)
2. **Confidence:** Code your confidence the BCT is present using + or ++ as indicated in BCTT training
3. **Target behaviour:** Code as D, P, S, A, M, other, U (for diet, physical activity, smoking, alcohol, medication adherence, other and list the behaviour, or if no specific behaviour mentioned use U for ‘unspecified’), or other. Only code ‘other’ if the BCT is targeting a behaviour directly linked to health outcomes (e.g. sleep-related behaviours, sexual health behaviours). Any BCTs around sedentary behaviour are categorised under ‘physical activity’.
4. **Mode of delivery:** The mode by which the facilitator delivers the BCT to the service user, NOT how the service user enacts the BCT. BCTs can be delivered via more than one mode, for example a video could be coded as both visual and auditory. Code as:
   1. Visual **[V]**: Delivered in a visual format (e.g. pictures, photographs, 3D models, behavioural demonstration, video, graphs, diagrams, symbols);
   2. Auditory/verbal **[A]**: Delivered in an audio format (e.g. facilitator speech, sound recording, music, round of applause)
   3. Text **[T]**: Delivered in a written format (e.g. patient handbook, pdf’s, email, messages, worksheets, text-based Powerpoint slides/videos)
   4. **U** for unclear
5. **Format of delivery:** Code how BCTs were delivered in the digital platform/digital materials. Can be coded as more than one format (e.g. a video sent via a one-to-one message in the app; a PDF document sent via email). Code as:
   1. Telephone **[T]:** Delivered via a health coach over the phone (e.g. initial assessment call)
   2. Email **[E]:** Content that is sent to service users’ email addresses
   3. Message **[M]:** Delivered via a chat function, one-to-one messaging, SMS messages, messaging via a group forum
   4. Push notifications **[P]:** Delivered via a ‘push’ notification, automated ‘nudge’ messaging, algorithmic messaging
   5. Video **[V]:** Content that is delivered via a video (this could be an information video, or a video message from a health coach)
   6. Written documents **[D]:** Content that is delivered via worksheets, handbooks, PDFs, etc.
   7. App **[A]:** Content that is delivered via the app (or website application)
   8. Quiz **[Q]:** Content that is delivered by a quiz (interactive activity). This quiz may be present in the app/web application, if so, code format as both A and Q
   9. External link **[L]:** Content that is included in the app or website application, but is delivered via an external link (e.g. to an app or website application)
6. **Deliverer of BCT:** [Code H for health coach, P for participant and leave blank for digital platform]**:**

- Health coach delivery is defined as the delivery of a BCT via the health coach (e.g. over the phone, via one-to-one messaging)
- Participant delivery is defined as the delivery of a BCT via a participant (e.g. in response to a question, or asking a question to the health coach). If the BCT is delivered by the participant *only*, record this on the coding sheet but highlight the row of the BCT so that researchers are aware of this during analyses
- Leave blank if the BCT is on the app, but has not been specifically stated by the health coach
- Digital platform delivery is defined as the delivery of a BCT that is *offered* via the digital platform (I.e., whether or not service users choose to take it up/engage with it)

1. **Group vs. individual delivery:** Leave blank for individual delivery as this will be by far the most common; code as G if a BCT is delivered in a group forum.
2. **Interactive vs. passive vs. prompted delivery:** [Code I for interactive, P for passive, PROMPTED for prompted, U for unclear]: Interactive is defined as expecting or requiring a response or action by the service user (e.g. asking the service user to set a goal, input tracking data, or practice a behaviour there and then for an exercise video); Passive is defined as delivery of a BCT that is imparted and received without requiring action or communication by the service user (e.g. providing information or instructions, advising the service user to practice the behaviour at home or sending nudge messages); Prompted is defined as a BCT that is prompted rather than directly delivered, regardless of whether the BCTTv1 definition specified that the BCT can be prompted (e.g. the definition in the BCTTv1 for Goal setting (behavior) was as follows: “Set or agree on a goal defined in terms of the behavior to be achieved” - however, this BCT was coded when patients were prompted to set a goal elsewhere as part of the intervention, for example, by clicking on the ‘Physical activity goal’ interactive tool)
3. **Location:**

- **For web pages/documents/transcripts:** Provide the page number as indicated in the document (e.g. p2), and the approximate quarter of the page in which the BCT appears (i.e. Q1, Q2, Q3, or Q4) – or provide a range of page/quarters if the BCT is described over more than one.
- **For emails/messages:** Provide the approximate quarter of the page in which the BCT appears (i.e. Q1, Q2, Q3, or Q4)
- **For recordings of telephone calls/videos:** Provide the exact time as indicated on the recording/video in which the BCT is mentioned (e.g. 17min, 46 secs), or provide a time range if this BCT is discussed over a period of time

1. **Evidence:** Briefly summarise the evidence indicating presence of the BCT. Use key original wording to retain meaning.
2. **Comments:** Make a note of any problems applying the BCT, uncertainties, unusual occurrences, etc.
3. **Coding rules:**

- For the BCT **‘Information about health consequences’** (directly linking behaviour and health outcome) code as a new BCT when interrupted by other activities (e.g. general information not comprising ‘info about health consequences’ or a separate subheading section) or when a new “level” of behaviour is targeted. E.g. different levels of dietary behaviours include: fruit and veg, fats, sugars, carbohydrates, proteins, etc. Different levels of physical activity behaviours include cardiovascular exercise, strength training, etc. Do not code new BCTs for each outcome e.g. “Eating less fat can help you **lower your cholesterol** and **lose weight**” would be coded once – this is to avoid coding multiple BCTs for long bullet pointed lists of outcomes for one behaviour/level of behaviour.
  - If the level of behaviour is not stated for this BCT, code as unspecified. E.g. code as ‘Diet (unspecified)’ when the statement is just talking about diet generally.
  - If there is more than one level of the target behaviour, code separately if they make sense as stand-alone statements. E.g. a list of health behaviours that reduce the risk of developing T2D: “eating less saturated fats, eating more fruit and veg…” – these would be coded as separate BCTs as they target different levels of dietary behaviour and they are delivered as separate statements.
  - If there is more than one level of target behaviour delivered in just one sentence and cannot be broken up into separate statements, code as one BCT only but list the different levels within that BCT. E.g. “If you eat too much of any of these foods [veg, carbs, protein] than your body needs, your weight will go up” – this would be coded as one BCT with the target behaviour and levels: ‘Diet (veg, carbs, protein).’ Differentiated instances of this BCT need to be stand-alone statements that would make sense on their own.
- For any other BCT, code as a new BCT when a new behaviour is targeted (e.g. diet, PA, etc.) or when participants start a new activity (e.g. new video, different sub-section within an article). The “levels” of behaviour do not need to be specified for any other BCT.
- For the BCT **‘Information about health consequences’** document whether the health consequence is experienced by the service user (e.g. weight gain, high blood pressure) by coding **‘Information about health consequences (a)’** and document whether the health consequence is a physiological response that is not experienced or measured (e.g. “your beta cells will get tired,” “the insulin locks will become rusty”) by coding **‘Information about health consequences (b).’**
- For the BCT **‘Social support (unspecified)’** code as a prompted BCT when signposted to other sources of social support e.g. another webpage providing information about a self-help group for the behaviour.
- For the BCT **‘Behavior substitution’** code as BCT when the unwanted behaviour and wanted/neutral behaviour are mentioned together and it’s implicitly implied that the wanted/neutral behaviour is intended to substitute the unwanted behaviour, but doesn’t necessarily explicitly prompt substitution. E.g. The text ““Try to eat lean and non-breaded versions of poultry, meat and fish.” Would **not** be coded as a BCT because it does not explicitly state the unwanted behaviour, whereas “Consider eating less carbohydrates and more vegetables and salad if you are trying to manage your weight” would be coded as a BCT because it explicitly states the unwanted (carbohydrates) and wanted/neutral behaviour (vegetables and salad).

Highlighted key:

Red = low confidence

Blue = external website

Yellow = prompted BCT

|  | **BCT code**  (from BCTT v1) | **BCT label**  (from BCTT v1) | **Confidence**  + or ++ | **Target behaviour**  D, P, S, A, M, U, other | **Mode of delivery**  V = visual  A = auditory  T = text  U = unclear | **Format of delivery**  T = telephone  E = email  M = message  P = push notification  V = video  D = written documents  A = app  Q = quiz  L = external link | **Deliverer of BCT**  H = health coach  P = participant  G = group  Blank = app | **Group vs. individual delivery**  G = group forum  Blank = individual | **Interactive vs. passive vs prompted delivery**  I = interactive  P = passive  PROMPTED = prompted  U = unclear | **Location**  (page, quarter/ minutes, seconds) | **Evidence of BCT**  (summary of BCT delivered) | **Comments** |
| --- | --- | --- | --- | --- | --- | --- | --- | --- | --- | --- | --- | --- |
| **INSERT ITEM NAME (ITEM TYPE, ITEM URL NUMBER) e.g. Kidney problems (Article, 1071)** | | | | | | | | | | | | |
|  |  |  |  |  |  |  |  |  |  |  |  |  |
|  |  |  |  |  |  |  |  |  |  |  |  |  |
|  |  |  |  |  |  |  |  |  |  |  |  |  |
|  |  |  |  |  |  |  |  |  |  |  |  |  |
|  |  |  |  |  |  |  |  |  |  |  |  |  |
|  |  |  |  |  |  |  |  |  |  |  |  |  |
|  |  |  |  |  |  |  |  |  |  |  |  |  |
|  |  |  |  |  |  |  |  |  |  |  |  |  |
|  |  |  |  |  |  |  |  |  |  |  |  |  |
|  |  |  |  |  |  |  |  |  |  |  |  |  |
|  |  |  |  |  |  |  |  |  |  |  |  |  |
|  |  |  |  |  |  |  |  |  |  |  |  |  |
|  |  |  |  |  |  |  |  |  |  |  |  |  |
|  |  |  |  |  |  |  |  |  |  |  |  |  |
|  |  |  |  |  |  |  |  |  |  |  |  |  |
|  |  |  |  |  |  |  |  |  |  |  |  |  |
|  |  |  |  |  |  |  |  |  |  |  |  |  |
|  |  |  |  |  |  |  |  |  |  |  |  |  |
|  |  |  |  |  |  |  |  |  |  |  |  |  |
|  |  |  |  |  |  |  |  |  |  |  |  |  |
