# Supplementary material for: Changes in a Digital Type 2 Diabetes Self-management Intervention During National Rollout: Mixed Methods Study of Fidelity
Source: J Med Internet Res. 2022 Dec 7;24(12):e39483. doi: 10.2196/39483 (PMC9773035; doi:10.2196/39483)
Supplement: Multimedia Appendix 3 [file jmir_v24i12e39483_app3.docx]

**Appendix 2.** Pre-specified list of self-management tasks

| **Task domain** | **Tasks** |
| --- | --- |
| Medical management | Medication |
|  | Physical activity |
|  | Smoking |
|  | Alcohol |
|  | Diet |
|  | Weight management |
|  | Foot care |
|  | Eye care |
|  | Self-monitoring |
|  | Working with health professionals |
|  | Managing acute complications |
|  | Sleep |
|  | Recreational drugs |
|  | Sexual health |
|  | Complementary medicine |
| Role management | Driving |
|  | Travel |
|  | Employment |
|  | Finances |
|  | Adopting a new identity or role |
|  | Social relationships and situations (with friends, family, colleagues or carers) |
|  | Social occasions |
|  | Holidays |
|  | Insurance |
|  | Eating out in restaurants, cafes or work canteens |
|  | Religion |
| Emotional management | Fear and anxiety |
|  | Sadness |
|  | Anger |
|  | Guilt |
|  | Denial |
|  | Stress |
|  | Shock |
|  | Self-confidence |
|  | Happiness |
|  | Cognitive functioning |
