# Supplementary material for: Changes in a Digital Type 2 Diabetes Self-management Intervention During National Rollout: Mixed Methods Study of Fidelity
Source: J Med Internet Res. 2022 Dec 7;24(12):e39483. doi: 10.2196/39483 (PMC9773035; doi:10.2196/39483)
Supplement: Multimedia Appendix 4 [file jmir_v24i12e39483_app4.docx]

**Appendix 4.** Kappa values for assessing inter-rater reliability of BCT coding

| **Web page name** | **Type of web page** | **Kappa Value** |
| --- | --- | --- |
| Benefits of physical activity | Video | 0.782541 |
| Emotional problems | Article | 1 |
| Lower carbohydrate plate | Article | 0.794702 |
| Activity routines and goals | Article | 1 |
| Physical activity goals you could use | Article | 0.656827 |
| Your diabetes review | Article | 0.491803 |
| SMART goals | Article | 0.661818 |
| Lawrence on the challenges of high blood sugar | Video | 1 |
| Challenges of taking medicines | Video | 0.656827 |
| Concerns about taking medicines: forgetting to take medicines | Article | 0.738764 |
| Measuring blood pressure at home | Article | 1 |
| Step 2 - Learn your early warning signs | Article | 0 |
| Blood pressure monitors | Article | 1 |
| Driving and low blood sugar | Article | 1 |
| Ways to increase your wellbeing: connect with other people | Article | 0.794702 |
| Fear and anxiety | Article | 0.794702 |
| Complications | Article | 1 |
| Making changes | Article | 0 |
| Eating when ill | Article | 0 |
| Eating at restaurants | Article | 0.661818 |
| Motor insurance | Article | 1 |
| Duncan on the challenges of walking to work | Video | 1 |
| Shift work | Article | 1 |
| Diabetes in the media | Article | 1 |
| Working with your diabetes team | Article | 1 |
| Recreational drugs | Article | 1 |
| If you already have a job | Article | 1 |
| How much do you know about type 2 diabetes? | Quiz | 1 |
| At the GP surgery | Article | 1 |
| Food diary | Tool | 0.903427 |

**Average kappa: 0.797931**
