# Supplementary material for: Changes in a Digital Type 2 Diabetes Self-management Intervention During National Rollout: Mixed Methods Study of Fidelity
Source: J Med Internet Res. 2022 Dec 7;24(12):e39483. doi: 10.2196/39483 (PMC9773035; doi:10.2196/39483)
Supplement: Multimedia Appendix 5 [file jmir_v24i12e39483_app5.docx]

**Appendix 5.** Instances of behaviour change techniques in Healthy Living for each health behaviour

| **Health behaviour** | **‘Learn’**  (273 pages) | ***First 60% of ‘Learn’***  *(138 pages) ^a^* | **‘Find answers’** (583 pages) | **‘Tools’**  (39 pages) | **Email communication** (20 messages) | **Healthy Living**  (All 895 pages) |
| --- | --- | --- | --- | --- | --- | --- |
| Diet | 154 | *92 (60%)* | 470 | 35 | 0 | **659** |
| Physical activity | 161 | *126 (78%)* | 279 | 27 | 4 | **471** |
| Medication adherence | 111 | *69 (62%)* | 326 | 17 | 0 | **454** |
| Alcohol | 57 | *30 (53%)* | 154 | 12 | 0 | **223** |
| Unspecified/multiple behaviours | 61 | *38 (62%)* | 86 | 19 | 5 | **171** |
| Smoking | 15 | *8 (53%)* | 74 | 0 | 0 | **89** |
| Other ^b^ | 9 | *0 (0%)* | 12 | 0 | 0 | **21** |

^a^ Percentage in brackets corresponds to the proportion of BCTs in the first 60% of the structured curriculum compared to the entire structured curriculum, rounded to the nearest whole number;

^b^ Includes sleep-related behaviour, sex-related behaviour, recreational drugs and non-prescribed medicines
