# Supplementary material for: Changes in a Digital Type 2 Diabetes Self-management Intervention During National Rollout: Mixed Methods Study of Fidelity
Source: J Med Internet Res. 2022 Dec 7;24(12):e39483. doi: 10.2196/39483 (PMC9773035; doi:10.2196/39483)
Supplement: Multimedia Appendix 6 [file jmir_v24i12e39483_app6.docx]

**Appendix 6.** Description of Healthy Living using the TIDieR framework

| **TIDieR item** *^a^* | **Healthy Living for people with type 2 diabetes Programme (Healthy Living)** |
| --- | --- |
| Why (Rationale) | The principal strategic drivers for this project were the NHS England Five Year Forward View [1] and to deliver the following Structured Education and Treatment target indicators:   - Diabetes patients who have achieved all of the NICE-recommended treatment targets (Three targets for adults-HbA1c, cholesterol and blood pressure: one target for children-HbA1c); - People with diabetes diagnosed less than a year ago who attend a structured education course.   The investment seeks to deliver on NHS England’s strategy to harness information and technology to support individuals to manage their care by developing a low-cost service to support individuals with T2DM. |
| What (Materials) | Healthy Living is a free digital NHS service for people living with type 2 diabetes to support them to self-manage their condition. The service can be used on a range of digital devices (i.e. smartphones, desktops, tablets). The website contains 895 web pages.  Website content is broken down into three main sections:   1. Learn (structured curriculum based on the HeLP-Diabetes: Starting Out structured course [2]) – 273 pages split into 26 modules; 2. Find answers (sections dedicated to different topics relating to T2DM where users could dip in and out of different pages and sections, based on the HeLP-Diabetes website [3]) – 583 pages split into 37 sections; 3. Tools (interactive tools) – 39 pages including goal setting, action planning and self-monitoring tools.   Content includes information about what type 2 diabetes is, its causes and how it can be managed and treated; behavioural advice on diet, physical activity, alcohol, smoking and medication adherence; and emotional and practical support. There are interactive tools for users to set and review goals and action plans for weight, diet, physical activity, daily steps, alcohol, medicines and wellbeing. There are also self-monitoring tools for weight, daily steps, diet and blood glucose.  Pre-existing interventions that are incorporated into Healthy Living, included DownYourDrink [4,5], POWeR [6,7], StopAdvisor [8,9], Living Life to the Full [10], and HealthTalkOnline personal video stories [11]. Users are also sent communication to promote engagement via emails and in-app notifications. No physical materials are offered to users. |
| What (Procedures) | Healthy Living is intended for people diagnosed with T2DM in England, carers and healthcare professionals, available by self-referral (there are ongoing plans to develop primary care referral, but no plans to provide facilitated access from a healthcare professional). Users register online by visiting the self-registration page of the website. Users can read articles; watch videos; complete self-assessment quizzes; set, plan and review goals and identify potential barriers to achieving these goals and ideas for navigating these barriers. Users can opt-in for notifications for step and weight tracking. Users can submit an online form for technical support or ring the support helpline number, but there are no health care professionals involved in supporting registration or use of the website. |
| Who provided | An external digital service provider was commissioned to develop and provide this NHS England service. NHS England assigned a project manager from the NHS England Diabetes Team to manage programme delivery according to the procurement contract. Healthy Living has NHS branding and gave the impression to users of being an NHS service. Technical support is provided by an administrator from the digital service provider to help people with technical problems such as lost passwords and to coordinate additional support from relevant teams within the service provider as required. Coaching or individual clinical advice is not offered in Healthy Living. |
| How (modes of delivery) | The service is delivered online for individuals. |
| Where | The service can be used wherever people have a digital device and internet access. |
| When and how much | Once registered, patients can use the service as much (or as little) as they wanted and could take as long as they wanted. However, patients are encouraged to use the structured ‘Learn’ curriculum. Modules in this curriculum can be completed in one sitting, or progress can be saved and users could resume at any time. NHS England specified that patients who progress through the first 60% of the ‘Learn’ curriculum are classified as having completed the programme. This threshold was agreed upon by the policy team based on benchmarking completion against other similar services and structured education programmes. |
| Tailoring | There is a small amount of tailoring based on questions after registering, which filters content relevant to the user’s smoking, employment and driving status. Written feedback is provided to users who complete the self-assessment quizzes, using validated tool calculations to provide responses based on their answers. These responses would indicate to the user any recommended actions the user might need to take to address their resulting score. This could include signposting to sections of the content or external services, suggestions of actions to take such as increasing physical activity or reducing alcohol consumption, and prompting setting goals in the programme to help achieve these recommended changes. |
| Modifications | The website is being developed on an ongoing basis to improve usability, accessibility and user engagement with the service. Modifications are completed in line with user research and feedback, and guidelines including the Government Digital Service (GDS) Standard [12], Web Content Accessibility Guidelines (WCAG) [13], Digital Technology Assessment Criteria [14] and NHS Digital Content [15] and Style Guidelines [16]. |

^a^ Items correspond to the headings from the TIDieR framework [17]

**References**

1. NHS England, Public Health England, Health Education England, Monitor, Care Quality Commission, NHS Trust Development Authority. Five Year Forward View. 2014.

2. Poduval S, Marston L, Hamilton F, Stevenson F ME. Feasibility, Acceptability, and Impact of a Web-Based Structured Education Program for Type 2 Diabetes: Real-World Study. JMIR Diabetes. 2020;5(1):e15744.

3. Murray E, Sweeting M, Dack C, Pal K, Modrow K, Hudda M, et al. Web-based self-management support for people with type 2 diabetes (HeLP-Diabetes): randomised controlled trial in English primary care. BMJ Open. 2017;7(9):e016009.

4. Linke S, McCambridge J, Khadjesari Z, Wallace P, Murray E. Development of a Psychologically Enhanced Interactive Online Intervention for Hazardous Drinking. Alcohol and Alcoholism 2008 Nov 1;43(6):669–674. doi: 10.1093/alcalc/agn066

5. Wallace P, Murray E, McCambridge J, Khadjesari Z, White IR, Thompson SG, Kalaitzaki E, Godfrey C, Linke S. On-line Randomized Controlled Trial of an Internet Based Psychologically Enhanced Intervention for People with Hazardous Alcohol Consumption. PLoS One Public Library of Science; 2011 Mar 9;6(3):e14740. doi: 10.1371/journal.pone.0014740

6. Yardley, L., Williams, S., Bradbury, K., Garip, G., Renouf, S., Ware, L., Dorling, H., Smith, E., & Little P. Integrating user perspectives into the development of a web-based weight management intervention. Clin Obes 2012;2(5–6):132–141. doi: 10.1111/cob.12001.

7. Yardley L, Ware LJ, Smith ER, Williams S, Bradbury KJ, Arden-Close EJ, Mullee MA, Moore M V, Peacock JL, Lean MEJ, Margetts BM, Byrne CD, Hobbs RFD, Little P. Randomised controlled feasibility trial of a web-based weight management intervention with nurse support for obese patients in primary care. International Journal of Behavioral Nutrition and Physical Activity 2014;11(1):67. doi: 10.1186/1479-5868-11-67

8. Michie S, Hyder N, Walia A WR. Development of a taxonomy of behaviour change techniques used in individual behavioural support for smoking cessation. Addictive Behaviors 2011;36(4):315–319. doi: 10.1016/j.addbeh.2010.11.016.

9. Brown J, Michie S, Geraghty AWA, Yardley L, Gardner B, Shahab L, Stapleton JA, West R. Internet-based intervention for smoking cessation (StopAdvisor) in people with low and high socioeconomic status: a randomised controlled trial. Lancet Respir Med 2014;2(12):997–1006. doi: 10.1016/S2213-2600(14)70195-X

10. Pittaway S, Cupitt C, Palmer D, Arowobusoye N, Milne R, Holttum S, et al. Comparative, clinical feasibility study of three tools for delivery of cognitive behavioural therapy for mild to moderate depression and anxiety provided on a self-help basis. Ment Health Fam Med. 2009;6(3):145–54.

11. Herxheimer A, McPherson A, Miller R, Chapple A, Shepperd S, Ziebland S SE. [DIPEx (Database of Individual Patients Experience of illness): a multimedia proposal to share experiences and information about illnesses between patients and health professionals]. Aten Primaria. 2003;31(6):386–8.

12. UK Government. Government Functional Standard GovS 005: Digital, Data and Technology. 2019. Available from: https://www.gov.uk/guidance/digital-data-and-technology-functional-standard-version-1

13. Web Accessibility Initiative. Web Content Accessibility Guidelines (WCAG) 2.1. 2018. Available from: https://www.w3.org/TR/WCAG21/

14. NHSX. The Digital Technology Assessment Criteria for Health and Social Care (DTAC). London; 2021.

15. NHS. Content style guide. Available from: https://service-manual.nhs.uk/content

16. NHS Digital. NHS Digital style guidelines. 2021. Available from: https://digital.nhs.uk/about-nhs-digital/corporate-information-and-documents/nhs-digital-style-guidelines

17. Hoffmann TC, Glasziou PP, Boutron I, Milne R, Perera R, Moher D, et al. Better reporting of interventions: template for intervention description and replication (TIDieR) checklist and guide. Res Methods Report. 2014;348:g1687.
